# Statinintoleranz und statinassoziierte Muskelschmerzen

**DOI:** 10.1007/s00059-022-05114-w

**Published:** 2022-04-22

**Authors:** Paulina Elena Stürzebecher, Friederike Schumann, Ursula Kassner, Ulrich Laufs

**Affiliations:** 1 Klinik und Poliklinik für Kardiologie, Universitätsklinikum Leipzig, 04103 Leipzig, Deutschland; 2 Medizinische Klinik für Endokrinologie, Diabetes und Ernährungsmedizin – Arbeitsbereich Lipidstoffwechsel, Charité – Universitätsmedizin Berlin, Campus Virchow-Klinikum, Berlin, Deutschland

**Keywords:** LDL-Cholesterin, Lipidsenkende Therapie, Hydroxymethylglutaryl-CoA-Reduktase-Inhibitoren, Unverträglichkeit, Kombinationstherapie, LDL cholesterol, Lipid-lowering therapy, Hydroxymethylglutaryl-CoA reductase inhibitors, Intolerance, Combination therapy

## Abstract

Statine gehören zu den bestuntersuchten Medikamenten. Aufgrund der umfangreichen Evidenz in Bezug auf Wirksamkeit und Sicherheit gehören sie zur Basis der lipidsenkenden Therapie. Während die Verträglichkeit von Statinen in den großen verblindeten Studien auf Placeboniveau liegt, stellt die sogenannte Statinintoleranz (SI) im klinischen Alltag ein häufiges und komplexes Problem dar. Am häufigsten werden statinassoziierte muskuläre Schmerzen (SAMS) berichtet. SI ist in vielen Fällen mit einer unzureichenden Senkung des LDL(„low-density lipoprotein“)-Cholesterins (LDL-C) assoziiert und erhöht damit das kardiovaskuläre Risiko. Die Diagnose von SAMS basiert auf dem Ausschluss möglicher alternativer Ursachen für Muskelsymptome und dem Ausschluss von Noceboeffekten durch eine diagnostische Strategie des Absetzens der Statinbehandlung, der Beobachtung und Bewertung der Symptome, gefolgt von einer erneuten Verabreichung eines anderen, zunächst niedrig dosierten Statins mit nachfolgender Dosissteigerung. Ein Großteil der Patienten mit SI und SAMS kann durch dieses Vorgehen Statine dauerhaft und ohne Beschwerden einnehmen. Bei unzureichender LDL-C-Senkung kommen frühzeitig Kombinationstherapien zum Einsatz. Es ist eine wichtige Aufgabe der verschreibenden Ärzte und aller an der Behandlung Beteiligten, durch eine geeignete Kommunikation die Einnahmetreue von Statinen zu erhöhen. Zahlreiche Fragen zu SI sind noch offen und werden u. a. in einem laufenden Register adressiert.

## Abstract (en)

Statins are among the best studied drugs. Due to the extensive evidence regarding efficacy and safety, they are the cornerstone of lipid-lowering therapy. While the tolerability of statins in large blinded studies is at the placebo level, so-called statin intolerance (SI) is a frequent and complex problem in everyday clinical practice. Statin-associated muscular pain (SAMS) is most commonly reported. In many cases SI is associated with inadequate lowering of low-density lipoprotein (LDL) cholesterol (LDL-C), thereby increasing the cardiovascular risk. The diagnosis of SAMS is based on the exclusion of possible alternative causes of muscular symptoms and the exclusion of nocebo effects through a diagnostic strategy of discontinuation of statin treatment, observation and assessment of symptoms, followed by renewed administration of a different statin initially at a low dose with subsequent dose increase. A large proportion of patients with SI and SAMS can take statins permanently and without discomfort by this approach. If LDL‑C lowering is insufficient, combination therapies are used. It is an important task of the prescribing physicians and all those involved in the treatment to increase the adherence to statins through appropriate communication. Numerous questions on SI remain open and are being addressed by an ongoing register.

## Einleitung

Statine reduzieren durch Senkung des Serum LDL(„low-density lipoprotein“)-Cholesterins (LDL-C) kardiovaskuläre Erkrankungen. Aufgrund des hervorragenden Wirkungs- und Sicherheitsprofils dieser Medikamentenklasse werden Statine auf dem Boden von Lebensstilempfehlungen in allen Leitlinien als Erstlinientherapie in der Primär- und Sekundärprävention von kardiovaskulären Erkrankungen mit hohem Evidenz- und Empfehlungsgrad bewertet [26].

Im Unterschied zu den randomisierten Studien gibt ein relevanter Anteil der Patienten im Alltag Beschwerden, insbesondere Muskelschmerzen, unter Statintherapie an. Nach aktueller Datenlage sind diese Beschwerden zu einem großen Teil, aber nicht alle, als Noceboeffekt zu werten. Die hohe Relevanz des Problems ergibt sich aus der Häufigkeit der berichteten Beschwerden sowie der assoziierten Reduktion der Einnahmetreue und der unzureichenden LDL-C-Senkung mit der Konsequenz eines höheren Risikos für kardiovaskuläre Ereignisse [8].

## Statine

Die Ära der Statine begann in den 1970er-Jahren. Die Arbeitsgruppe um den japanischen Mikrobiologen A. Endo erforschte Pilze, welche cholesterinhemmende Stoffe zum Schutz vor Mikroben produzieren. Ihnen gelang 1976 die Isolation des ersten HMG(Hydroxy-3-Methylglutaryl)-CoA(Coenzym A)-Reduktase-Hemmers namens Mevastatin (ML-236B) aus dem Schimmelpilz *Penicillium citrinum*. Aufgrund hepatozellulärer Toxizität wurde Mevastatin jedoch nicht weiter verfolgt [10]. Hofmann und Kollegen konnten schließlich 1979 aus *Aspergillus terrus* das Präparat Lovastatin isolieren, welches dann 1987 als erstes Statin auf dem US-amerikanischen Markt zugelassen wurde [25].

Statine inhibieren die HMG-CoA-Reduktase, welche den geschwindigkeitsbestimmenden Schritt der Cholesterinsynthese vermittelt. Statine binden reversibel und kompetitiv an die HMG-CoA-Reduktase und blockieren hierdurch kompetitiv die Andockstelle für HMG-CoA [25]. In der Folge werden die Umwandlung von HMG-CoA in Mevalonat und damit die endogene Cholesterinbiosynthese reduziert. In den Leberzellen führt dies zu einer Steigerung der Expression des LDL-C-Rezeptors. Hierdurch wird die Cholesterinaufnahme aus dem Blut erhöht.

Der Wirkmechanismus ist bei allen Statinen gleich, wobei die verschiedenen Statinpräparate aufgrund ihrer chemischen Struktur unterschiedliche pharmakologische und pharmakodynamische Eigenschaften aufweisen (Tab. 1). Diese Unterschiede bestehen u. a. hinsichtlich Metabolisierung, Wasserlöslichkeit und LDL-senkender Potenz [24].

**Table 1:** 

–	Rosuvastatin	Atorvastatin	Simvastatin	Lovastatin	Pravastatin	Fluvastatin
*Prozentuale LDL-C-Senkung*	–	–	–	–	–	–
10–20 %	–	–	5 mg	10 mg	10 mg	20 mg
20–30 %	–	–	10 mg	20 mg	20 mg	40 mg
30–40 %	5 mg	10 mg	20 mg	40 mg	40 mg	80 mg
40–45 %	5–10 mg	20 mg	40 mg	80 mg	80 mg	–
45–50 %	10–20 mg	40 mg	*80 *mg (nicht empfohlen)*	–	–	–
50–55 %	20 mg	80 mg	–	–	–	–
56–60 %	40 mg	–	–	–	–	–
*Herkunft*	Synthetisch	Synthetisch	Fungal	Fungal	Fungal	Synthetisch
*Metabolismus: CYP*	2C9 + 2C19	3A4	3A4	3A4	–	2C9
*Halbwertszeit bis Elimination*	20 h	14 h	3 h	1,1 h	1,8 h	3 h

*CYP* Cytochrome P450, primär abbauendes Cytochromsystem der Leber

Die neuen synthetischen Statine Atorvastatin und Rosuvastatin haben die höchste Anzahl an Bindungsstellen an der HMG-CoA-Reduktase und damit eine hohe Bindungsaffinität [25]. Zudem haben sie eine lange Halbwertszeit. Sie senken daher das LDL‑C effektiver und sind potenter als andere Statine. Der Metabolisierungsweg von Atorvastatin, Simvastatin und Lovastatin über Cytochrom P450 3A4 (CYP3A4) sollte in Bezug auf Medikamenteninteraktionen berücksichtigt werden. Inhibitoren von CYP3A4, wie manche Antimykotika, Antibiotika und Immunsuppressiva, erhöhen die Bioverfügbarkeit und damit das Nebenwirkungspotenzial dieser Statine [24]. Mittlerweile sind alle Statine als Generika erhältlich.

## Studienlage zur Wirksamkeit von Statinen

Es gibt viele randomisierte, kontrollierte Studien zu Statinen mit großen Fallzahlen und langer Nachbeobachtungsphase. Insgesamt kann auf über 30 Jahre Studienerfahrung zurückgegriffen werden. Die durch die Cholesterol Treatment Trialists’ (CTT) Collaboration durchgeführte Metaanalyse von 14 dieser Studien mit einer Fallzahl von 90.056 und einem Follow-up von 5 Jahren zeigte, dass eine Statintherapie das Risiko für schwerwiegende vaskuläre Ereignisse um etwa 25 % pro 1 mmol/l (~40 mg/dl) Senkung des LDL‑C pro Jahr verringert [4]. Der absolute Nutzen einer Statintherapie hängt vom kardiovaskulären Risiko der Person, von der absoluten LDL-C-Senkung und der Therapiedauer ab. Eine Senkung des LDL‑C um 2 mmol/l mit einem Statin (z. B. Atorvastatin 40 mg täglich, derzeitige Kosten ca. 4 € pro Monat) über einen Zeitraum von 5 Jahren bei 10.000 Patienten verhindert im Mittel das Auftreten schwerwiegender kardiovaskulärer Ereignisse bei etwa 1000 Patienten, also bei 10 % (= absoluter Nutzen), in der Sekundärprävention und bei 500 (5 %) in der Primärprävention [9]. Frauen und Männer sowie Patienten mit und ohne Diabetes profitieren gleichermaßen [16, 32]. Die Risikoreduktion für den einzelnen Patienten richtet sich nach dem individuellen vaskulären Risiko, der Höhe des Ausgangscholesterins, dem Ausmaß der LDL-C-Senkung und der Dauer der Therapie [24]. Auch bei Senioren und Hochbetagten reduzieren Statine das kardiovaskuläre Risiko analog zu Jüngeren [7, 12]. Die Risikoreduktion besteht auch dann, wenn Statine im höheren Alter neu verordnet werden [30]. Das Absetzen einer Statintherapie bei Patienten über 75 Jahre in der Primärprävention ist mit einem um 33 % höheren kardiovaskulären Risiko assoziiert [13].

In Patientengruppen, die ein höheres Risiko haben, an einer nichtvaskulären Ursache zu sterben (z. B. Patienten mit Herzinsuffizienz mit reduzierter Pumpfunktion oder terminaler Niereninsuffizienz), reduzieren Statine ebenfalls vaskuläre Ereignisse (insbesondere Myokardinfarkte und Schlaganfälle). Dies reicht aufgrund der Heterogenität der Todesursachen in diesen Studienkollektiven jedoch nicht aus, um einem Effekt auf die Gesamtmortalität zu sehen [11, 36].

Statine als Medikamentenklasse sind generell sicher und gut verträglich. Unerwünschte Arzneimittelwirkungen (UAW), die auf eine Statintherapie zurückzuführen sind, sind Myopathien (definiert als Muskelschmerzen oder -schwäche in Verbindung mit einem starken Anstieg der CK(Kreatininkinase)-Konzentration im Blut; [2]). Das relative Risiko für einen Anstieg von Hämoglobin A_1c_ (HbA_1c_) auf einen Wert über 6,5 ist bei Patienten mit Prädiabetes um 10 % erhöht [33]. Bei einer Behandlung von 10.000 Patienten über 5 Jahre mit einem wirksamen Regime (z. B. Atorvastatin 40 mg täglich) treten etwa 5 Fälle von Myopathie (von denen 1 Fall bei Fortsetzung der Statintherapie zur Rhabdomyolyse fortschreitet) und 50 bis 100 neue Fälle von Diabetes mellitus (definiert als HbA_1c_ > 6,5 %) auf. Das absolute Risiko von UAW bleibt im Vergleich zum absoluten Nutzen gering [9]. Dies gilt insbesondere für Patienten mit Prädiabetes.

In der CTT-Metaanalyse traten mehr als 6000 nichtvaskuläre Todesfälle auf. Es gab keinen Hinweis, dass die Senkung der LDL-C-Konzentration durch eine Statintherapie eine Auswirkung auf nichtvaskuläre Todesursachen (z. B. Neoplasien) hatte [4, 9]. In Schwangerschaft und Stillzeit sind Statine nicht zugelassen, da sie hier nicht getestet wurden. Eine erhöhte Teratogenität ergibt sich aus experimentellen Studien, Registern oder Fallserien nicht [15].

Anhand der Studienlage gibt es keinen Anhalt für eine Auswirkung der Statintherapie auf Gedächtnisleistung/kognitive Funktion, Kataraktentwicklung und Nierenfunktion [19]. Die Daten zeigen keinen Hinweis auf relevante hepatotoxische Wirkungen von Statinen. Schwere Leberschädigungen treten nur sehr selten auf (ca. 1 von 100.000). Daher empfiehlt die aktuelle ESC(European Society of Cardiology)/EAS(European Atherosclerosis Society)-Leitlinie keine routinemäßige Kontrolle der Transaminasen [26]. Gerade Patienten mit metabolischem Syndrom und Steatosis hepatis, die häufig erhöhte Transaminasen aufweisen, profitieren von der Statintherapie [24]. Die umfangreichen Erkenntnisse aus randomisierten Studien aus einem Beobachtungszeitraum von über 3 Jahrzehnten machen es unwahrscheinlich, dass weitere schwerwiegende Nebenwirkungen von Statinen bisher unentdeckt geblieben sind [9].

## Hinweise für die Praxis

Der erste Schritt der lipidsenkenden Therapie sind Lebensstilmaßnahmen, insbesondere Nichtrauchen, körperliche Aktivität und eine ausgewogene Ernährung. Aufgrund der klaren Evidenz zur Wirksamkeit und Sicherheit stellen Statine die Eckpfeiler der medikamentösen Maßnahmen dar [24].

Vor Therapiebeginn ist die Bestimmung der Lipoproteine einschließlich einer einmaligen Bestimmung von Lipoprotein (a) (Lp(a)), CK und Alaninaminotransferase (ALT) zu empfehlen. Die Effekte der LDL-C-Senkung sind bereits nach 7 bis 10 Tagen zu beurteilen. Zur Kontrolle des Therapieansprechens ist eine LDL-C-Bestimmung etwa 3 bis 4 Monate nach Therapiebeginn sinnvoll. Eine Verlaufskontrolle der CK ist nur bei Myalgien indiziert [24].

Die hepatische Cholesterinsynthese ist bei Nahrungskarenz erhöht, sodass die abendliche Einnahme von Statinen etwas stärkere Effekte erzielen kann. Dies betrifft v. a. die Statine fungalen Ursprungs (Simvastatin, Lovastatin, Pravastatin). Durch die lange Halbwertszeit der neuen synthetischen Statine Atorvastatin und Rosuvastatin rückt dieser Effekt in den Hintergrund. Der mit Abstand wichtigste Faktor für die LDL-C-Senkung ist die Medikamenteneinnahmetreue. Daher sollte derjenige Einnahmezeitpunkt gewählt werden, der die größtmögliche Einnahmetreue des Patienten ermöglicht [3]. Da im Mittel die Medikamenteneinnahmetreue morgens am besten ist, empfehlen die Autoren lang wirksame Statine mit morgendlicher Einnahme.

Eine hoch intensive Statintherapie kann das LDL‑C um mehr als 50 % senken. Eine Therapie mit mittlerer Intensität ist definiert als die Dosis, die den LDL-C-Wert voraussichtlich um 30–50 % senkt. Zu beachten ist, dass es interindividuelle Unterschiede bei der LDL-C-Senkung mit der gleichen Dosis eines Medikaments gibt [26]. Personen mit einer hohen intestinalen Cholesterolabsorption sprechen weniger stark auf Statine an und profitieren frühzeitig von einer Ergänzung um Ezetimib.

Eine Dosisverdopplung von Statinen bringt aufgrund der kompetitiven Hemmung der HMG-CoA-Reduktase nur eine verhältnismäßig geringe weitere LDL-C-Senkung von etwa 6 %. Aufgrund der synergistischen Wirkung ist daher die Kombination mit Ezetimib effektiver (weitere 20–30 % LDL-C-Senkung) bei meist besserer Verträglichkeit. Der Trend geht hin zu einer individuell basierten Kombinationstherapie verschiedener lipidsenkender Medikamente in niedriger Dosierung [24]. Es sind keine schädlichen Wirkungen von sehr niedrigen LDL-C-Konzentrationen (z. B. < 1 mmol/l [40 mg/dl]) bekannt [26].

## Statinintoleranz

### Prävalenz, Definition SAMS

Ein Großteil der kardiovaskulären Hochrisikopatienten erreicht weiterhin nicht die LDL-C-Zielwerte [22]. Ursächlich hierfür sind in vielen Fällen ein frühzeitiges Absetzen der Statintherapie und eine geringe Therapieadhärenz aufgrund von statinassoziierten Nebenwirkungen, insbesondere Muskelsymptomen [6]. Eine reduzierte Einnahmetreue von Statinen ist linear mit einer erhöhten Sterblichkeit vergesellschaftet und damit von großer Relevanz [8]. Die Definition von SI in der Literatur ist nicht einheitlich. Die National Lipid Association (NLA) definiert SI als die Unverträglichkeit von mindestens 2 Statinen entweder aufgrund von unerwünschten Symptomen oder auffälliger Laboranalysen, die in zeitlichem Zusammenhang mit der Statinbehandlung stehen. Nach Absetzen des Statins sind die Symptome reversibel und durch einen erneuten Versuch reproduzierbar. Andere bekannte Faktoren sind auszuschließen [19]. Statinassoziierte Muskelsymptome (SAMS) sind die häufigste Ursache bei statinintoleranten Patienten, sodass im Folgenden der Fokus hierauf gelegt wird.

### Studienlage

In beobachtenden Registern und in der klinischen Praxis bestehen bei bis zu einem Drittel der Patienten SAMS. In großen randomisierten Studien dagegen liegt die Rate deutlich geringer und auf Placeboniveau [16, 28, 32].

Es gibt damit eine auffällige Diskrepanz zwischen der Inzidenz von SAMS im klinischen Alltag oder in Beobachtungstudien und den Ergebnissen randomisierter, placebokontrollierter Studien (RCT). Gründe hierfür sind u. a. das Fehlen einer Kontrollgruppe bei Beobachtungsstudien und die Tatsache, dass Muskelschmerzen nicht systematisch erfasst wurden, sodass die Inzidenz tendenziell in Registern überschätzt wird [9]. Es ist zu betonen, dass durch die hohe Anzahl von RCT mit vielen verschiedenen Patienten und unterschiedlichen Einschlusskriterien zuverlässige und valide Daten erhoben wurden, die in den klinischen Alltag übertragen werden können. Die Vergleichbarkeit der Ergebnisse der verschiedenen RCT macht es unwahrscheinlich, dass in der veröffentlichten Literatur eine wesentliche Verzerrung vorliegt [9].

Die STOMP(Effects of Statins on Muscle Performance)-Studie, eine randomisierte, doppelblinde, placebokontrollierte, prospektive Studie, die speziell zur Untersuchung der Wirkung von Statinen auf die Skelettmuskulatur konzipiert wurde, ergab keinen Anhalt für eine Schädigung der Muskulatur durch Statine. Nach 6 Monaten Therapie mit hoch dosiertem Atorvastatin (80 mg) wurden Muskelschmerzen bei 9,4 % der Statinbehandelten und bei 4,6 % der Kontrollprobanden detektiert. Ein Unterschied bei der Messung der Muskelkraft oder der körperlichen Leistungsfähigkeit zwischen mit Statin und mit Placebo behandelten Patienten wurde nicht festgestellt [31].

In der SAMSON-Studie (Design: *n* = 1 [Einzelpatientenstudie]) wurden statinintolerante Patienten jeweils 4 Monate mit einem Statin und 4 Monate mit einem Placebo behandelt und blieben 4 Monate ohne Therapie. Beschwerden innerhalb dieses Jahres wurden von den Patienten täglich dokumentiert. 90 % der vom Patienten angegebenen SAMS konnten nicht kausal auf das Statin zurückgeführt werden und wurden durch das reine Einnehmen einer Tablette ausgelöst, d. h. stellten einen Noceboeffekt dar. Bei rund 10 % der dokumentierten Beschwerden gab es eine Korrelation mit der Statintherapie [18]. Muskuläre Beschwerden sind häufig, und die Daten sprechen dafür, dass in den meisten Fällen die Statineinnahme nicht ursächlich für die muskulären Beschwerden ist. Die Daten zeigen jedoch auch, dass bei einem geringen Prozentanteil eine statinassoziierte Beschwerdesymptomatik besteht. Die Größenordnung von 90 % Nocebo- und 10 % statininduzierten SAMS deckt sich mit den Ergebnissen weiterer Studien (ASCOT, StatinWISE; [14, 17]). Eine aktuelle Metaanalyse von 176 Studien mit 4 Mio. Personen bestätigt eine Prävalenzrate der SI von etwa 9 % [5]. Bei einer übereinstimmend geschätzten Prävalenz der SAMS von 5–10 % betrifft diese Thematik bei der weiten Verbreitung der Statintherapie mehrere 100.000 Patienten allein in Deutschland [23]. Es handelt sich also um ein relevantes klinisches Problem.

### Vorgehen bei Statinintoleranz in der Praxis

Ein belastbarer laborchemischer, genetischer oder bildmorphologischer Test, der zur Diagnosestellung und Quantifizierung von SAMS angewendet werden kann, steht nicht zur Verfügung. Daher ist eine operationale Diagnostik erforderlich, die auf einer ausführlichen Anamnese und dem zeitlichen Zusammenhang der Beschwerden mit Beginn, Pause und Reexposition von Statinen beruht. Die CK-Aktivität ist nicht sensitiv und spezifisch für SAMS [26]. Zudem ist zu beachten, dass eine erhöhte CK-Konzentration *per se* nicht schmerzhaft ist [20]. Rosenson und Kollegen haben einen Score zur Unterstützung der Erkennung von SAMS (Statin-Associated Muscle Symptom Clinical Index [SAMS-CI]) entwickelt [34]. Die klinische Präsentation von SAMS ist jedoch ausgesprochen heterogen und reicht von Verspannung und Steifheit über Schmerzen bis hin zu Krämpfen. Eine einheitliche Definition gibt es nicht. SAMS gehen typischerweise ohne oder nur mit einer geringen CK-Erhöhung einher, sind symmetrisch in den proximalen Extremitäten lokalisiert und beginnen etwa 4 bis 6 Wochen nach Beginn der Therapie. Typisch ist eine Besserung der Beschwerden innerhalb von 2 Wochen nach Absetzen des Statins [35]. Mögliche Risikofaktoren für SAMS sind u. a. weibliches Geschlecht, hohes Alter, verschiedene Komorbiditäten und Polypharmazie (Tab. 2, [5]).

**Table 2:** 

Risikofaktoren	
Anthropometrisch	Alter > 80 Jahre
	Weibliches Geschlecht
	Untergewicht/Übergewicht
	Hohe sportliche Aktivität
Komorbiditäten	Akute Infektion/Trauma
	Endokrinologische Ursachen (z. B. Diabetes mellitus, Schilddrüsenfunktionsstörungen)
	Renale/hepatische Funktionsstörungen
	HIV
	Depression
Genetik	Genetische Faktoren wie Polymorphismen, die für Cytochrom-P450-Isoenzyme oder Medikamententransporter kodieren (eine genetische Diagnostik aufgrund fehlender klinischer Relevanz wird derzeit nicht empfohlen)
Entsprechende Anamnese	Unerklärte Muskelschmerzen/CK-Erhöhung in der Vergangenheit
	Statinassoziierte Muskelsymptome unter anderem Statin
	Neuromuskuläre Erkrankung
Pharmakokinetik	Hochdosisstatintherapie
	Polypharmazie
	Medikamenteninteraktion
	Ausgeprägter Alkohol- bzw. Pampelmusensaftkonsum, Drogen

*HIV* humanes Immundefizienzvirus, *CK* Kreatininkinase

**Figure 1:**
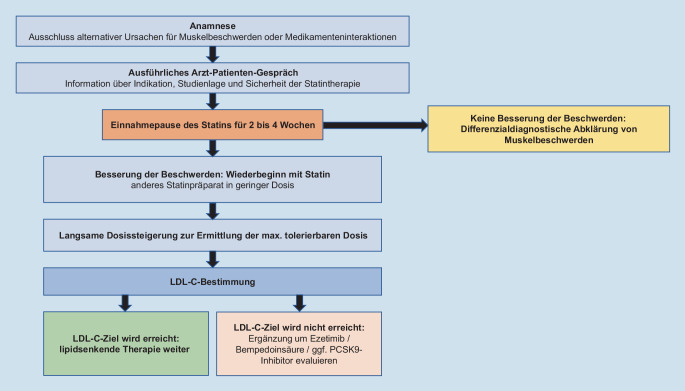

Das konkrete Vorgehen in der Praxis ist in Abb. 1 dargestellt.

Der Schlüssel im Umgang mit SI ist, sich Zeit für die Patienten zu nehmen. Durch eine umfangreiche Anamnese können die meisten alternativen und sekundären Ursachen der berichteten Beschwerden ausgeschlossen werden.

Eine Einnahmepause von 2 bis 4 Wochen sollte zum „washout“ des Statins erfolgen. Bei Persistenz der Beschwerden sind differenzialdiagnostische Ursachen wie orthopädische Erkrankungen zu klären. Auch neurologische, endokrinologische und systemische inflammatorische Ursachen oder Durchblutungsprobleme sollten bei entsprechender Klinik bedacht werden [23].

Kommt es in der Einnahmepause zu einer Verbesserung der Beschwerden, sollte ein anderes Statin in einer zunächst niedrigen Dosis etabliert werden. Eine Möglichkeit ist, auch ein lang wirksames Statin zunächst umtägig einzunehmen. Bei Nichterreichen des LDL-C-Zielbereichs kommen Kombinationstherapien mit der maximal verträglichen Statindosis (Ezetimib, Bempedoinsäure) und/oder alternative LDL-C-senkende Therapeutika (PCSK9[Proproteinkonvertase Subtilisin/Kexin Typ 9]-Hemmer) zum Einsatz. Eine frühzeitige Kombinationstherapie, z. B. mit Ezetimib, kann gute Ergebnisse bei guter Verträglichkeit erzielen [35]. Zusätzlich ist mit Bempedoinsäure ein weiteres orales Medikament zur Lipidsenkung auf dem deutschen Markt erhältlich. Pharmakologisch wird die Substanz als Prodrug eingenommen, welches in der Muskulatur nicht wirksam ist, da das aktivierende Enzym nur in der Leber exprimiert wird [21]. Sollte mit einer oralen Kombinationstherapie keine ausreichende Senkung erreicht werden, ist die Indikation für eine PCSK9-Inhibitor-Therapie mittels monoklonaler Antikörper oder der neuen siRNA („small inhibiting ribonucleic acid“) Inclisiran zu evaluieren [26].

Für Nahrungsergänzungsmittel zur Linderung von Muskelsymptomen (Vitamin D, Coenzym Q10) existiert kein randomisierter Wirksamkeitsbeleg. Bei berichteten positiven Effekten in Einzelfällen könnte es sich um Placeboeffekte handeln, daher werden Nahrungsergänzungsmittel von den Fachgesellschaften nicht empfohlen [35]. Dagegen konnte in einer aktuellen Studie gezeigt werden, dass Patienten mit und ohne Beschwerden unter Statintherapie gleichermaßen von körperlicher Betätigung profitierten. Sport erhöhte nicht die Rate an Muskelschmerzen, sondern steigerte die Lebensqualität der Teilnehmer [1].

Die molekularen Ursachen vom SAMS sind weitgehend unverstanden. Diskutiert werden eine Verminderung der mitochondrialen Funktion sowie eine Veränderung des Muskelproteinabbaus und des zellulären Energiestoffwechsels. Es besteht eine Assoziation zwischen Polymorphismen im Gen des hepatischen Transporters SLCO1B1 („solute carrier organic anion transporter family member 1B1“) und simvastatininduzierten SAMS. Diese genetische Variante führt zu einer erhöhten Konzentration von Statinen im Blut und bedingt ein erhöhtes Risiko für Muskelschmerzen. Der Einfluss der Genvariante auf die Plasmakonzentration verschiedener Statine ist sehr heterogen und in abnehmender Reihenfolge wie folgt verteilt [23]: Simvastatin, Atorvastatin, Pravastatin, Rosuvastatin, Fluvastatin.

Aufgrund fehlender klinischer Konsequenz ist eine genetische Untersuchung bei SAMS nicht indiziert. Hohe Dosierungen von Simvastatin sind generell nicht zu empfehlen.

Essenziell im Umgang mit statinintoleranten Patienten ist ein ausführliches Gespräch. Viele Patienten sind durch mögliche schwerwiegende Nebenwirkungen, die in den Medien diskutiert werden, verunsichert. Gleichzeitig sind all diejenigen Personen, bei denen eine Statintherapie ein kardiovaskuläres Ereignis verhindert hat, nicht präsent in der Berichterstattung, sodass eine verzerrte Wahrnehmung entsteht. In einer dänischen Studie wurde eine Assoziation zwischen negativer Berichterstattung und frühzeitigem Abbruch der Statintherapie berichtet [29]. Ein gutes Arzt-Patienten-Gespräch zur Indikation der Statintherapie, zum Nutzen und zu einem möglichen Noceboeffekt kann die Therapieadhärenz steigern. Mögliche Nebenwirkungen von Statinen sind reversibel, die Folgen eines Herzinfarkts oder Schlaganfalls hingegen nicht. Jeder Patient, der SAMS angibt, benötigt Aufmerksamkeit und Zeit – nicht aus Sicherheitsgründen, sondern um die Therapietreue zu unterstützen und die LDL-C-Ziele zu erreichen.

Große Fallserien zeigen, dass über 90 % der Patienten mit SAMS langfristig doch ein Statin tolerieren [27, 37]. Auch in der oben erwähnten Einzelpatientenstudie (SAMSON) konnten 50 % der initial als statinintolerant beschriebenen Patienten ein Statin dauerhaft einnehmen, was die Notwendigkeit einer guten Aufklärung betont [18]. Es ist eine wichtige ärztliche Aufgabe, die Einnahmetreue von Statinen zu verbessern, um die kardiovaskuläre Morbidität und Mortalität zu reduzieren.

**Figure 2:**
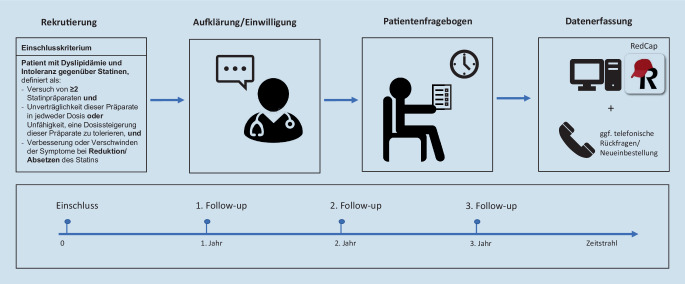

Es verbleiben weiterhin viele offene Fragen in Bezug auf das Patientenkollektiv mit statinassoziierten Nebenwirkungen. Sowohl die Ursachen als auch Möglichkeiten zur Prävention und zur Behandlung sowie der klinische Verlauf sind weitgehend unbekannt. Hier bedarf es u. a. beobachtender Studien, um Charakteristika, Versorgung und Prognose prospektiv zu untersuchen. Ein prospektives multizentrisches Register soll daher systematisch und prospektiv Patienten mit SI in Deutschland untersuchen (ClinicalTrials.gov Identifier: NCT04975594). Neben klinischen Daten werden auch Informationen zum psychosozialen Hintergrund und zur Medikamentenwahrnehmung der Patienten erhoben. Eine Nachverfolgung ist für 3 Jahre vorgesehen (Abb. 2). Ziel ist es, langfristig optimierte Strategien für die betroffenen Patienten aufzeigen zu können.

## Fazit für die Praxis

- Statine sind sicher und gut verträglich.
- Sie erreichen durch eine Senkung des LDL(„low-density lipoprotein“)-Cholesterins (LDL-C) eine Reduktion der kardiovaskulären Mortalität und Morbidität – auch im hohen Alter.
- Statinintoleranz und speziell statinassoziierte Muskelschmerzen (SAMS) führen zu niedriger Einnahmetreue, zur Einschränkung der Lebensqualität und erhöhen das kardiovaskuläre Risiko.
- Da die genauen molekularen Mechanismen von SAMS unbekannt sind, gibt es keinen spezifischen diagnostischen Nachweis. Die Bestimmung der Kreatininkinase (CK) ist nicht sensitiv oder spezifisch, da die überwiegende Mehrheit der SAMS nicht mit CK-Erhöhung einhergeht. In einer sorgfältigen Anamnese sollte neben Abklärung alternativer Ursachen auf die typische Präsentationsform, den zeitlichen Verlauf in Assoziation mit der Statineinnahme und das Auftreten von SAMS bei Reexposition nach Einnahmepause geachtet werden.
- Ein Großteil der statinintoleranten Patienten kann nach einer sorgfältigen Aufklärung und Anleitung langfristig mit Statinen behandelt werden.
- Durch eine individualisierte Kombinationstherapie ist es möglich die LDL-C-Zielwerte mit guter Verträglichkeit zu erreichen.
